# Contraceptive content shared on social media: an analysis of Twitter

**DOI:** 10.1186/s40834-024-00262-2

**Published:** 2024-02-07

**Authors:** Melody Huang, Alba Gutiérrez-Sacristán, Elizabeth Janiak, Katherine Young, Anabel Starosta, Katherine Blanton, Alaleh Azhir, Caroline N. Goldfarb, Felícita Kuperwasser, Kimberly M. Schaefer, Rachel E. Stoddard, Rajet Vatsa, Allison A. Merz-Herrala, Deborah Bartz

**Affiliations:** 1 Harvard Medical School, 25 Shattuck Street, 02115 Boston, MA USA; 2 Department of Biomedical Informatics, Harvard Medical School, 10 Shattuck Street, Suite 514, 02115 Boston, MA USA; 3 Department of Obstetrics and Gynecology, Brigham and Women’s Hospital, 75 Francis Street CWN-3, 02115 Boston, MA USA; 4 Department of Social and Behavioral Sciences, Harvard TH Chan School of Public Health, 677 Huntington Ave, 02115 Boston, MA USA; 5 Harvard-MIT Program in Health Sciences and Technology, Harvard Medical School, 77 Massachusetts Ave, 02139 Cambridge, MA USA; 6 Harvard PhD Program in Health Policy, 14 Story Street, 02138 Cambridge, MA USA; 7 Department of Obstetrics, Gynecology and Reproductive Sciences, University of California, San Francisco, 2356 Sutter Street, 94115 San Francisco, CA USA

**Keywords:** LARC, Birth control pill, SARC, Tweets, Contraceptive decision making, Contraceptive side effects, Social networks

## Abstract

**Background:**

Information on social media may affect peoples’ contraceptive decision making. We performed an exploratory analysis of contraceptive content on Twitter (recently renamed *X*), a popular social media platform.

**Methods:**

We selected a random subset of 1% of publicly available, English-language tweets related to reversible, prescription contraceptive methods posted between January 2014 and December 2019. We oversampled tweets for the contraceptive patch to ensure at least 200 tweets per method. To create the codebook, we identified common themes specific to tweet content topics, tweet sources, and tweets soliciting information or providing advice. All posts were coded by two team members, and differences were adjudicated by a third reviewer. Descriptive analyses were reported with accompanying qualitative findings.

**Results:**

During the study period, 457,369 tweets about reversible contraceptive methods were published, with a random sample of 4,434 tweets used for final analysis. Tweets most frequently discussed contraceptive method decision-making (26.7%) and side effects (20.5%), particularly for long-acting reversible contraceptive methods and the depot medroxyprogesterone acetate shot. Tweets about logistics of use or adherence were common for short-acting reversible contraceptives. Tweets were frequently posted by contraceptive consumers (50.6%). A small proportion of tweets explicitly requested information (6.2%) or provided advice (4.2%).

**Conclusions:**

Clinicians should be aware that individuals are exposed to information through Twitter that may affect contraceptive perceptions and decision making, particularly regarding long-acting reversible contraceptives. Social media is a valuable source for studying contraceptive beliefs missing in traditional health research and may be used by professionals to disseminate accurate contraceptive information.

## Background

 Contraceptive users rely on their social networks for information; patients often weigh the contraceptive experiences and attitudes expressed by informal sources, including friends and family, more heavily than information from health professionals [1, 2]. Social media is an increasingly important component of peoples’ social networks [3]. Nearly 90% of adults aged 18 to 29 regularly use at least one social media platform [4]. People also use social media for health information. For example, individuals turn to YouTube for information on gynecologic and sexual health [5], and to Reddit for information regarding sexually transmitted infection (STI) diagnoses [6]. Content posted on social media serves as a source of information for clinicians and patients to better understand peoples’ experiences with miscarriage [7], menopause [8], lupus and pregnancy decision making [9], and emergency contraception [10]. Importantly, social media influences health behaviors. A survey of teens and young adults found that information previously received from social media more closely correlated with contraception use at time of last intercourse compared with messaging from family, traditional media, and school [11]. Randomized control trials of social media interventions led to improved knowledge of contraceptives [12] and an increased preference for more reliable long-acting reversible contraceptives (LARCs) [12, 13]. 

Twitter is a free, text-based social networking platform with over 330 million monthly users [14]. Up to 46% of U.S. Twitter users use it at least daily [15]. The majority of Twitter users are 18–34 years old [16], similar to Instagram users [17], whereas Facebook users include an older demographic [18]. The population of Twitter users is representative of the gender and racial distribution within the U.S. [19] but is more likely to have higher education levels and incomes than the average U.S. adult [20]. Twitter is the most popular social media site for obtaining news and current events, with 55% of Americans regularly getting news from Twitter [21]. The unique 280-character limit leads to quick and succinct exchanges of information, and the ability to repost others’ tweets, or “retweet,” distinguishes Twitter from other popular social media platforms as a hub where users frequently engage in discussion regarding current events or popular topics.

 Twitter has been recognized as an important data source for health-related topics [22–24], including reproductive health [9, 10]. Popular U.S. media outlets have been shown to tweet about women’s health topics with a particular focus on contraception [25]. We previously used computer-generated natural language processing to analyze the sentiment expressed in over 600,000 tweets relating to contraceptive methods as positive, neutral, or negative [26]. However, an assessment of the specific content of tweets related to contraception has yet to be performed. Given the prevalence of contraception-related posts on Twitter, we conducted an exploratory content analysis of tweets about modern reversible, prescription contraceptive methods. Secondarily, we explored how this content changed over time, the sources of contraceptive tweets, the degree to which tweets solicited or provided advice, and the amplification of contraceptive content through tweet likes, replies (comments made on the tweet by other Twitter users), and retweets.

## Methods

Methods for tweet collection are detailed in a previous study on trends in attitudes toward contraceptive methods on Twitter [26]. Briefly, we generated a database of tweets using the Python library GetOldTweets3, which used 112 key words to search for all publicly available, English-language tweets related to reversible, prescription contraceptive methods that were posted between March 21, 2006 (founding of Twitter) and December 1, 2019 (open-source code: https://github.com/hms-dbmi/contraceptionOnTwitter). Tweets were not confined to the U.S. Keywords included brand, generic, and colloquial names and abbreviations of the contraceptive methods of interest: LARC methods (intrauterine devices [IUD] and Implanon/Nexplanon implants) and short-acting reversible contraceptive (SARC) methods (oral contraceptive pill [OCP], contraceptive patch, vaginal ring, and depot medroxyprogesterone acetate [DMPA] shot). The algorithm removed duplicate tweets, tweets in which the keyword was in the username (e.g., @NuvaRingLawyer), and tweets related to male or emergency contraceptives. For purposes of the parent study, tweets mentioning more than one contraceptive method were also excluded.

From this database, we performed simple random sampling to extract a random subset of 1% of all tweets posted between January 2014 and December 2019 to perform an exploratory content analysis. We chose this time frame to capture content from the majority of tweets (nearly 70% of all tweets about contraceptive methods were posted during this period) while still having enough tweets per year to meaningfully compare content over time. Since the number of tweets in the random sample mentioning the contraceptive patch was less than 200, we deliberately oversampled tweets about the patch to ensure thematic saturation. To ensure anonymity, we processed tweets to remove quotes, hashtags, URLs, and certain symbols to avoid association back to the original post. From each tweet, we extracted the text, the year the tweet was posted, and the number of likes, replies, and retweets associated with the tweet. Demographics of the tweet creators could not be assessed as accounts could be created with minimal, false, or misleading information. This study was determined to be non-human subjects research by the Harvard Medical School Institutional Review Board.

In order to explore the content topics of tweets dedicated to contraceptive methods, we created a codebook generated from themes that emerged from the tweets to guide our analysis. One researcher (MH) conducted a first-pass annotation of a random subset of 500 tweets, identifying main themes that emerged. These themes were developed into codes with specific coding definitions that fit within larger categories, making up the codebook (Appendix 1). To validate the codebook, two researchers (DB, AS) used the first draft to independently review another random sample of 100 tweets. Differences were discussed with a larger group (MH, DB, AS, EJ) to revise the codebook. This process was repeated once more until consensus was reached based on high agreement for each coding variable.

We assessed 36 codes (Appendix 1) that fell under three main categories: (1) tweet content topic, which referred to aspects of a contraceptive method that the tweet addressed, such as side effects, efficacy, etc. (2) tweet source, which identified if the tweet was posted by a contraceptive consumer, someone who knows a contraceptive consumer, or an official health or news source, or if no source could be determined; and (3) information solicitation or advice, which determined if the tweet was explicitly requesting information or providing advice about a method. Codes within tweet content topic were not mutually exclusive, and content topic was not mutually exclusive with information solicitation or advice (i.e. a tweet could be explicitly providing advice about a method’s side effects and would thus be coded under “side effects” and “providing advice”).

Each tweet was annotated by at least two reviewers. We first reviewed tweets for false positivity; if a tweet was not about the female contraceptive method or did not contain enough information with which to discern its meaning, it was excluded. To assess interrater reliability of classification of the tweet, Kappa-generated statistics ranged from 0.58 to 0.78 for most mutually exclusive variables, demonstrating moderate to substantial interrater reliability after the first round of analysis. A third reviewer (MH or KY) then reviewed each discrepancy to adjudicate differences. If the third annotation was discrepant from both initial reviewers’ annotations, the tweet was discarded from analysis. We recorded the number of likes, replies, and retweets a tweet received as a measure of its virality. Descriptive analyses were reported including frequencies and proportions.

## Results

The Python library extracted 989,627 English-language tweets that referenced at least one contraceptive method. After excluding duplicate tweets, tweets in which the keyword was in the username, and tweets mentioning male or emergency contraceptives, 838,739 tweets remained. After excluding tweets mentioning more than one contraceptive, 665,064 tweets remained, of which 457,369 were posted between January 2014 and December 1, 2019. Of this population, we took a random sample of 1%, rounding up to 4,600 tweets. In this sample, 2,455 tweets mentioned the IUD, of which less than 200 specified the IUD type. Thus, we combined tweets from all three IUD categories (copper IUD, levonorgestrel IUD, and unspecified type) into one general “IUD” category. We deliberately added 139 patch tweets to make a total of 200 patch tweets and then excluded 305 false positive tweets. Ultimately, 4,434 tweets (2,317 total IUD [2,130 unspecified type IUD, 112 copper IUD, 75 hormonal IUD], 583 shot, 576 implant, 516 OCP, 250 ring, and 192 patch) met criteria for final analysis.

**Fig. 1:**
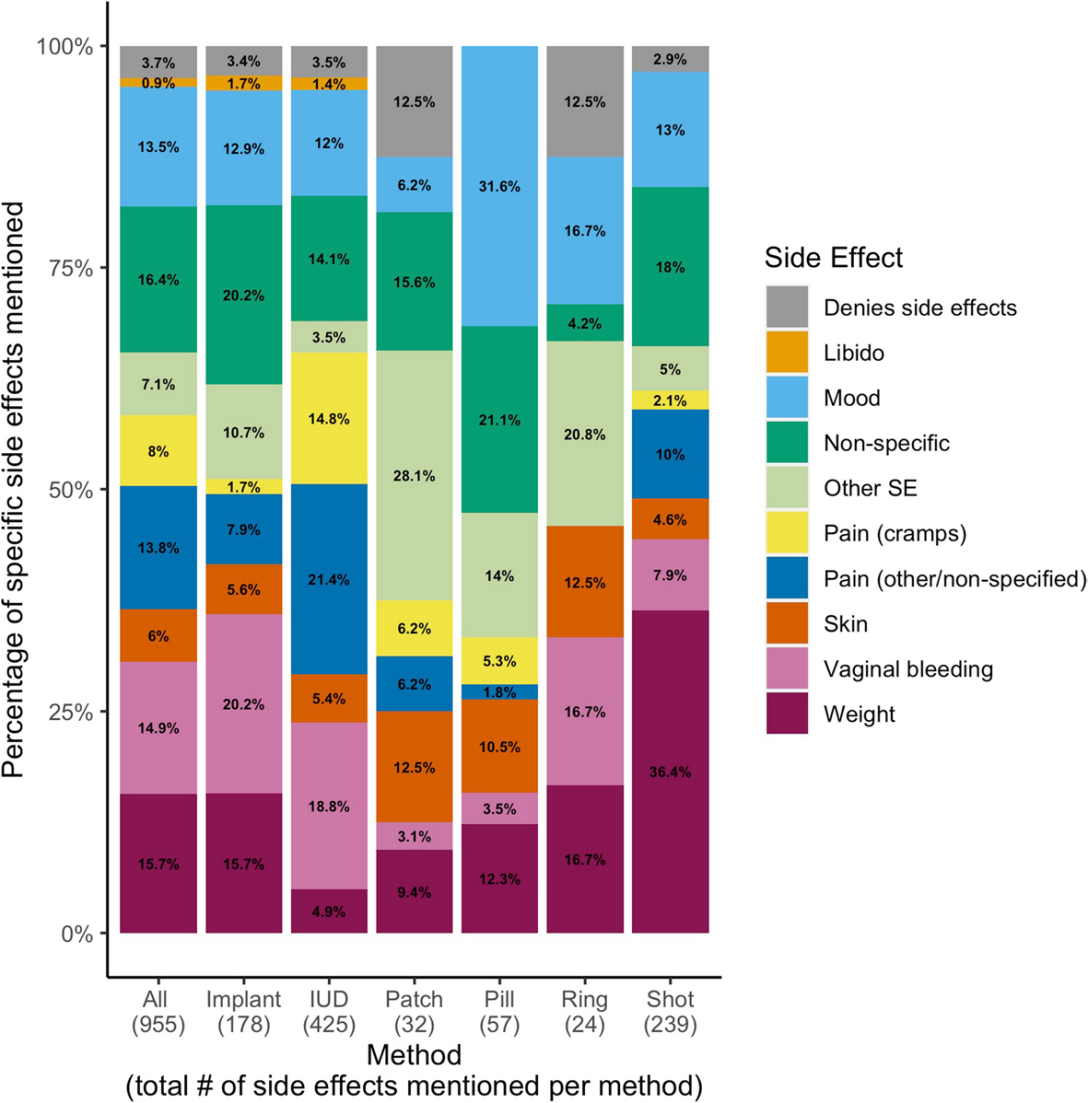
Proportion of Specific Side Effects Mentioned within Tweets by Contraceptive Method.  IUD, intrauterine device. OCP, oral contraceptive pill.  Proportion of tweets mentioning each side effect by all side effects mentioned for each contraceptive method. Tweets mentioning multiple side effects were scored as belonging to each specific side effect category once, meaning that a single tweet could contribute to multiple categories

Tweets reported in the text are direct quotations, and the coded source of the tweet is identified directly after the quote. Table 1 summarizes the distribution of content topics found within tweets by contraceptive method. Method decision making was the most frequently discussed content topic overall (*n* = 1139, 26.7% of total tweets) and within the subgroup of LARC methods (*n* = 648, 29.1% of IUD tweets; *n* = 217, 38.7% of implant tweets). This content topic included tweets about discontinuing, starting, or switching to a method; generalized statements about one’s experience on the method; and describing a general decision making process regarding method choice. Many tweets suggested that information from non-clinical sources influenced an individual’s decision making (“*I’ve heard of too many horror stories with the iud to ever wanna get it. i’ll leave it up to god lmao”*; source: contraceptive consumer). Side effects was the second most frequently discussed content topic overall (n = 874, 20.5% of total tweets) and the most frequently discussed content topic for the DMPA shot (n = 225, 40.1%). Weight was the most frequently mentioned side effect of the shot (n = 87, 36.4%) (Fig. 1).

**Table 1:** Content topics mentioned within tweets by contraceptive method

Content Topic	All methods (*n* = 4,266)^a^	IUD (*n* = 2,228)^a^	Implant (*n* = 560)^a^	Patch (*n* = 494)^a^	OCP (*n* = 494)^a^	Ring (*n* = 239)^a^	Shot (*n* = 561)^a^
**Method decision making**	1,139 (26.7)	648 (29.1)	217 (38.8)	44 (24.2)	43 (8.7)	44 (18.4)	143 (25.5)
**Side effects**	874 (20.5)	385 (17.3)	162 (28.9)	31(17.0)	49 (9.9)	22 (9.2)	225 (40.1)
**Uncategorized**	442 (10.4)	215 (9.7)	40 (7.1)	31 (17.0)	71 (14.4)	65 (27.2)	21 (3.7)
**Logistics of use/adherence**	397 (9.3)	102 (4.6)	30 (5.4)	46 (25.3)	95 (19.2)	42 (17.6)	83 (14.8)
**LARC placement/removal or DMPA administration**	396 (9.3)	257 (11.5)	87 (15.5)	N/A	N/A	N/A	52 (9.3)
**Efficacy (pregnancy)**	364 (8.5)	250 (11.2)	32 (5.7)	6 (3.3)	27 (5.5)	15 (6.3)	34 (6.1)
**News/research/ads**	262 (6.1)	99 (4.4)	19 (3.4)	19 (10.4)	79 (16.0)	21 (8.8)	25 (4.5)
**Access/cost**	261 (6.1)	185 (8.3)	15 (2.7)	8 (4.4)	37 (7.5)	5 (2.1)	11 (2.0)
**Politics/ethics**	252 (5.9)	179 (8.0)	10 (1.8)	1 (0.6)	54 (10.9)	0 (0)	8 (1.4)
**Safety/adverse events**	214 (5.0)	123 (5.5)	18 (3.2)	1 (0.6)	50 (10.1)	13 (5.4)	9 (1.6)
**Efficacy (bleeding patterns)**	134 (3.1)	80 (3.6)	17 (3.0)	3 (1.7)	16 (3.2)	5 (2.1)	13 (2.3)
**Mechanism of action**	103 (2.4)	68 (3.1)	8 (1.4)	4 (2.2)	15 (3.0)	3 (1.3)	5 (0.9)
**Sexual event**	84 (2.0)	54 (2.4)	2 (0.4)	2 (1.1)	4 (0.8)	18 (7.5)	4 (0.7)
**Healthcare interaction**	73 (1.7)	46 (2.1)	9 (1.6)	0 (0)	7 (1.4)	1 (0.4)	10 (1.8)
**Efficacy (non-specified)**	60 (1.4)	29 (1.3)	7 (1.3)	6 (3.3)	8 (1.6)	4 (1.7)	6 (1.1)

IUD, intrauterine device. OCP, oral contraceptive pill. LARC, long-acting reversible contraceptive. DMPA, depot medroxyprogesterone acetate. N/A, not applicable. Data are n (%) unless otherwise specified. Percentages in each column do not add up to 100 because one tweet can have more than one content topic, and content topics were counted independently. Content topics in which tweets made up < 1% of total tweets were excluded, including efficacy unrelated to pregnancy or vaginal bleeding patterns, STIs, and drug interactions

^a^Tweets with at least one content topic

Logistics of use or adherence (LOU) (9.3% of total tweets) tweets ranged from 14.8 to 25.3% for the SARC methods, and from 4.6 to 5.4% for the LARC methods. OCP tweets regarding logistics of use discussed misplacing the pill or difficulty with adherence (“*i feel so accomplished when i don’t forget a single bc pill all month*”; source: contraceptive consumer). Tweets about LARC placement or removal often expressed feeling fear (“*i was so scared about getting that implanon out of my arm*”; source: contraceptive consumer) or pain associated with the procedures. Fewer tweets discussed how their experiences were less difficult than expected (“…*people complain about how painful iud insertions are but i got mine on lunch and went back to working my shift. i’m literally fine??*”; source: contraceptive consumer). Tweets discussing a method’s efficacy in preventing pregnancy made up 11.2% of IUD tweets and 3.3–6.3% of other methods’ tweets. Most of these tweets focused on how the most effective methods are still imperfect (“*there is still 1% women who get pregnant with an iud”*; source: inconclusive). The “uncategorized” content topic, which contained humor, was the most common topic among tweets about the ring, likely due to the many jokes made about this method (“*if you like it, then you should’ve put a nuvaring on it*”; source: inconclusive).

**Fig. 2:**
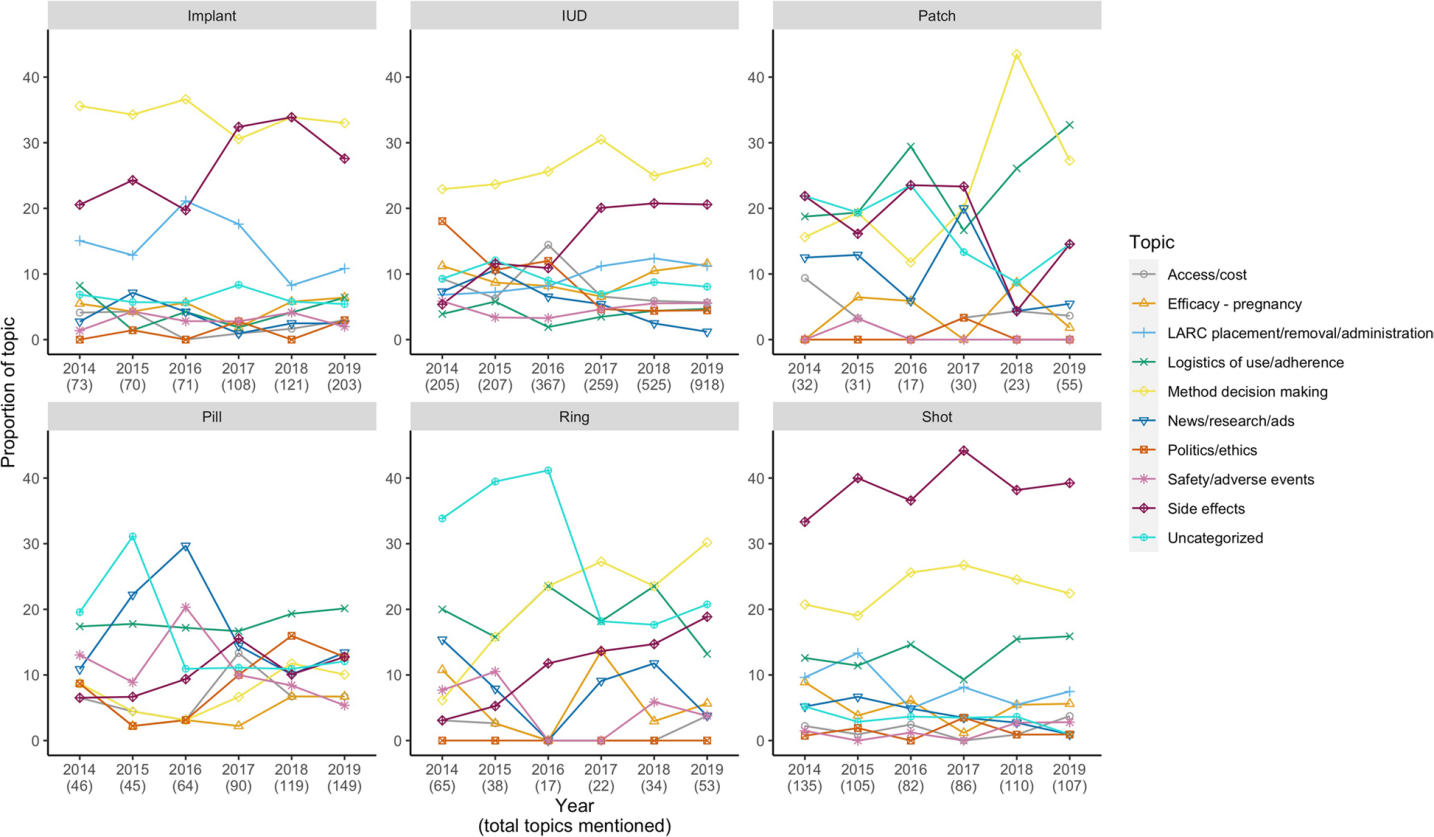
– Proportion of Content Topics Over Time by Contraceptive Method.  IUD, intrauterine device. OCP, oral contraceptive pill. LARC, long-acting reversible contraceptive. DMPA, depot medroxyprogesterone acetate.  Proportions were obtained by dividing the number of tweets mentioning each content topic by the total number of mentions of content topics per method and per year (given in parenthesis below each year). Tweets discussing multiple content topics were scored as belonging to each content topic category once, meaning that a single tweet could contribute to multiple categories. Content topic mentions of categories comprising <5% of total tweets (such as mechanism of action, as expressed in Table  1 ) were excluded for visual clarity

Figure 2 shows the proportions of content topics mentioned by method from 2014 to 2019. Most notably, the proportion of tweets mentioning LARC decision making remained high over time (30.6–36.6% of Implant tweets, 22.9–30.5% of IUD tweets). There was also an increase in the proportion of tweets discussing LARC side effects between 2016 and 2017, and side effects made up the largest proportion of DMPA tweets across the years.

Given the small number of tweets specifying the IUD type, we combined all IUD tweets into one category when coding. However, with the different side effect profiles of hormonal and copper IUDs, we performed a qualitative review of tweets specifying IUD type to assess for different themes between the two groups. Copper IUD tweets frequently discussed its non-hormonal mechanism as a positive attribute (“*I got the copper iud and I love it no hormones at all and didn’t even hurt at all!*”; source: contraceptive consumer). Copper IUD tweets also commonly mentioned heavy bleeding, which was frequently negative (“*I had the paraguard for a year. Ended up having it removed because my period got so bad that I could barely move for a week every month. I hated it so much*”; source: contraceptive consumer), but other times tolerable (“*i had the non hormonal iud for about 3 years. it made my periods much worse, but it was bearable. i did get pregnant on it which is super rare, but overall i say it’s worth it*”; source: contraceptive consumer). Hormonal IUD tweets noted minimal bleeding, which was usually desirable (“*the mirena iud has the added benefit of light (or absent) periods!*”; source: contraceptive consumer), but also spotting, which was undesirable. Overall, there were a number of both positive and negative tweets posted about each type.

We did not perform a systematic analysis on the prevalence of misinformation, as it was difficult to categorize for many tweets sharing personal experiences. However, we did come across tweets claiming inaccurate information about a method as fact (“…*an iud had a chance at causing future fertility issues*”; source: inconclusive). Multiple tweets about the efficacy of a method at preventing pregnancy (*n* = 4) espoused medically inaccurate information (“*the pill is the most effective form of contraception at 99% followed by condoms at 98%*”; source: inconclusive). There were also tweets that discussed sexism related to birth control use (“*anyway i’m going to get an iud and my doctors like take a few advil you’ll be chill and imagine if we told men that*.”; source: contraceptive consumer). A few tweets were outwardly misogynistic, particularly in sexually shaming women (“*i know the bitches that been taking the birth control shot for hella years prolly not gone be able to have kids in the future. shit fucked you up in the long run cause you ain’t wanna just keep yo legs closed and use a condom*”; source: vicarious consumer).

There were 4,335 tweets (97.8%) that had an agreed-upon code for “source.” Of these, 50.6% (*n* = 2,193) were posted by a contraceptive consumer (including former and potential users), and 8% (*n* = 347) were posted about others’ use of the contraceptive, such as relatives (“*my sis has gotten accidentally pregnant for both her kids. the second happened despite her iud*”) or public figures (“*youtube beauty blogger says her iud gave her devastating cystic acne*”). Six percent (*n* = 256) appeared to be posted by an official news or healthcare source. Tweets from a news or journalistic source were more frequent than tweets from a healthcare professional or organization. Some tweets included phrases such as “study shows,” suggesting they were news headlines (“*new: previous oral contraceptive use associated with better outcomes in patients with ovarian cancer, study finds*”). Others explicitly stated that the poster was a healthcare professional (“*as a pharmacist seeing old commercials for nuvaring, i want to stop filling those prescriptions because the ads are ridiculous*”). Many journalistic tweets highlighted complications associated with birth control methods (“*a massive study tracking 1 million women over a 13 year period has linked the contraceptive pill to depression*”), with fewer discussing potential benefits of these methods (“*iuds may cut risk of cervical cancer by a third, study indicates–- the guardian uk*”). Tweets from news sources also aimed to provide education about contraceptive methods, often quoting actual health professionals (“*gynecologists debunk myths surrounding the contraceptive pill acne, weight gain, moodiness, cancer–- daily mail*”). Tweets from a healthcare source were frequently from an organization providing birth control education or services (“*thought about getting an iud? We just expanded our capacity, so call today to make an appt. (416-961-0113)*”).

Only 6.2% (*n* = 276) of tweets explicitly requested information about a contraceptive method (by method, from 5.1% of IUD to 10.9% of patch tweets), whereas 4.2% (*n* = 184) explicitly provided advice about the method (from 1% of OCP to 6.3% of Implant tweets). Of tweets providing advice, 52.2% (*n* = 96) recommended a method, 38.6% (*n* = 71) recommended against a method, and 9.2% (*n* = 17) provided general advice without recommending for or against a method. More tweets recommended the IUD than cautioned against it (*n* = 58, 61.1% vs. *n* = 25, 26.3%, respectively), whereas more tweets cautioned against the shot than recommended it (*n* = 27, 75% vs. *n* = 9, 25%, respectively).

Of the 4,434 tweets, 43.5% (*n* = 1928) had at least one like, 23.1% (*n* = 1024) had at least two likes, and 15.7% (*n* = 696) had at least three likes. Average number of likes in total was 15.8, for IUD was 28.7, and for all other methods was under 2. Tweets about the IUD were most popular with 47.8% of IUD tweets having at least one like, followed by 42.1% of ring and 39.2% of pill tweets. Average number of replies on a tweet was 0.7, with 35.5% (*n* = 1574) of tweets having one or more replies. Average number of retweets was 2.6, with 12.1% (*n* = 536) of tweets having one or more retweets.

## Discussion

Our study assessed the content of a large random sample of tweets about modern, reversible contraceptive methods posted over six years. These tweets were most often posted by contraceptive users and most frequently discussed method decision making and side effects, particularly regarding LARC methods and the shot. Tweets about other SARC methods focused more on logistics of use or adherence. Tweets about the IUD were most popular in terms of likes. A small proportion of tweets explicitly requested information from or provided advice to other Twitter users.

Our findings support the social nature of contraceptive decision making that has been well-reported in the literature. We found that LARC tweets most frequently discussed method decision making, which adds to prior reports that LARC method choice is specifically impacted by social networks. In one study, Hispanic and African American patients more frequently reported not trusting their healthcare providers and seeking advice from social circles when deciding on LARC [27]. Individuals have also previously reported obtaining negative information about IUDs from their social networks [28]. 

LARC tweets more frequently discussed method decision making than OCP tweets, even though more U.S. individuals were using an OCP than a LARC during our study period [29, 30]. Given the more recent increase in LARC use, prior reports showed that LARCs were perceived as new and untested and were thus met with incorrect but firmly held beliefs about their efficacy [31, 32]. On the contrary, people had accepted OCP as the norm and continued to use it despite prior experiences of poor efficacy or side effects due to mistrust of newer longer-acting contraceptive methods [31–33]. It is possible that familiarity with the OCP led to less deliberation regarding its method choice on Twitter, whereas the increasing popularity of and skepticism towards LARCs resulted in greater discussion regarding its use.

Side effect profile is often a determining factor for method initiation or discontinuation and was the second most frequently discussed content topic [32, 34]. Social networks have often shaped individuals’ perceptions of side effects, which has led to avoidance or discontinuation of a method [2, 3]. Many tweets in our analysis supported this finding, implying that a specific side effect would determine one’s choice of the method. Notably, less than 4% of all side effect mentions denied the presence of a side effect. This may be explained by negativity bias: people are more prone to report feelings of dissatisfaction [35]. 

While weight gain is a commonly cited side effect of DMPA use, prior studies have reported menstrual disturbances as more common than weight changes [36–41] and more likely to lead to discontinuation of the shot [39–43]. However, while the number of overall side effect tweets about weight and menstrual irregularities is similar, we found more DMPA tweets about weight than any other side effect, suggesting people may care more about weight changes related to DMPA than prior reports have indicated. It is possible that individuals feel more comfortable sharing about weight online, which has the option of being anonymous, as opposed to sharing directly with interviewers in more traditional study formats. Furthermore, posting about weight digitally may feel less personal than verbalizing these ideas to another person, even if not anonymous. People may also feel more prone to discuss a vulnerable topic within their social circle online than with a researcher they do not know.

Our findings have several implications for healthcare professionals. It is notable that official news or healthcare sources posted such a small fraction of the tweets identified, and that even within that source group, most were posted by journalistic sources as opposed to identifiable healthcare providers. There may be room for healthcare workers to amplify their presence on social media to improve contraceptive education. Prior reports have suggested that incorporating technology may make contraceptive counseling more effective and convenient [44]. In particular, a study on patient attitudes toward Bedsider.org, a website providing contraceptive patient education, showed that individuals preferred reading about others’ personal experiences over generalized educational materials and appreciated the ability to ask their own questions and get them answered online [45]. Healthcare providers may consider utilizing the quick and interactive platform provided by Twitter to share contraceptive information through direct exchanges with social media users. In particular, they could harness the popular and frequent discussions surrounding LARCs on Twitter to answer questions and address misconceptions about these methods. This may be especially relevant for adolescents and young adults, who have the highest rates of social media use and lowest rates of LARC use [15, 29, 30]. 

Furthermore, it has been reported that people often enter healthcare visits with biases about contraceptives obtained from social networks [46, 47] and ultimately choose a method based on recommendations from social circles rather than from their physicians [48]. Despite these reports, providers do not regularly ask about information gained from social networks, even though doing so can positively encourage contraceptive, particularly LARC, use [46, 47]. One study showed that discussions about social influence on contraceptives are usually initiated by the patient, and that when providers do ask, they use close-ended questions that limit deeper conversations [49]. Our findings that individuals share and seek personal contraceptive experiences on Twitter further argue for clinicians to ask patients about contraceptive information gleaned from social networks, including social media. Questions should ideally be open-ended and can inquire about topics frequently discussed on Twitter, such as others’ experiences with LARCs or side effects (particularly with regards to weight changes), or social connotations associated with particular methods. While Twitter may provide valuable insight, with more tweets being created by personal contraceptive users than official healthcare sources, the available information may vary in reliability. Asking patients about information from social media can help reaffirm to patients the importance of social networks in contraceptive decision making while also addressing misconceptions to improve contraceptive counseling.

Our findings also have future research implications. While not an aim of our study, we recognized numerous tweets sharing misinformation. While methodologically difficult, a systematic analysis of the accuracy of information shared on Twitter would be a helpful addition to the literature. It would also be important to assess which type of contraceptive content on Twitter is most effective for influencing an individuals’ choice of contraceptive. Future research may use methods including network science to examine the ways in which Twitter users respond to and utilize the contraceptive information online and how that may impact clinical care.

By studying information posted on a public platform, we likely captured ideas of individuals who may be less represented in traditional health research. This is particularly important as people of racial and ethnic minority backgrounds have higher rates of unintended pregnancies but are less likely to be participants in traditional health research [50, 51]. Furthermore, we eliminated reporting bias by studying ideas that individuals organically shared on social media.

This study has several limitations. First, we analyzed each tweet as an independent entity (without including preceding tweets in a chain or those in response). This made it sometimes difficult to determine the intention or context surrounding a post, leaving room for some personal interpretation by reviewers. Second, we only characterized English-language tweets, so our results may not capture perceptions of populations who speak a language other than English. Furthermore, Twitter users have been shown to have higher education levels and incomes than the average U.S. adult, and the ideas of individuals who have a greater social media presence may be overrepresented, so our results may not be generalizable to a wider population. We were unable to make strong conclusions about the different side effect profiles or method choice discussions between the different IUD types because the majority of IUD tweets did not specify the type. Finally, in excluding tweets that mentioned more than one contraceptive method, we may have missed attitudes held by Twitter users when comparing multiple forms of contraception.

## Conclusions

Individuals are exposed to a variety of contraceptive topics through Twitter, which varied by contraceptive method. This social media content may contribute to contraceptive decision making. Clinicians should be aware that information on social media may affect patients’ contraceptive choices. Social media is a valuable source for studying contraceptive perceptions and experiences that patients bring into the exam room. A better understanding of contraceptive information available on social media and its impact on patients may motivate health professionals to use this platform to disseminate accurate contraceptive information.

## Appendix

### Codebook

**Table:** 

Content Topic*	**Description**	**Example tweet**
Access/cost	Tweet addresses the access to or cost of contraception.	*agh my dad's insurance doesn't cover my iud?? trying to figure out if my work insurance will cover it in two months when i switch.*
Drug interactions	Tweet addresses interactions between contraceptive method and other medications.	*hi.. i was born because my mothers antibiotics made her oral contraceptive not work. Helllooooooooo*
Efficacy (bleeding patterns)	Tweet addresses the role of the contraceptive method in improving irregularities or pain associated with vaginal bleeding.	*he's put me on the contraceptive pill to try and jolt the periods into being more regular/stop the bleeding.*
Efficacy (pregnancy)	Tweet addresses the efficacy of contraceptive method in preventing pregnancy.	*the contraceptive pill is the most effective form of contraception which is 99% effective followed by condoms at 98%.*
Efficacy (non-specified)	Tweet addresses the general efficacy of the contraceptive method without specifying.	*many people have found a single medication that works for them. it's similar to mental health medications, sometimes there's only one thing or one type of thing that works for you. for some people that's birth control or iuds.*
Efficacy (other)	Tweet addresses the efficacy of contraceptive method in treating another specified condition (acne, mood, etc) that is not related to preventing pregnancy or regulating vaginal bleeding.	*acne a's skin update u.s. fda approves first and only oral contraceptive demonstrated to treat …*
Healthcare interaction	Tweet focusing on experiences with healthcare providers or medical institutions.	*i’ve been super lucky that my current ob/gyn takes my pain very seriously but i still wound up having an iud and part of my colon removed*
LARC placement/removal or DMPA administration	Tweet addresses the process of placing or removing an IUD or implant or administering the DMPA shot.	*getting my implanon birth control removed is considered minor surgery. i was given two doses of novacaine and i bled so much. ugh.*
Logistics of use/adherence	Tweet addresses the logistics of use of a contraceptive method or adherence/non-adherence to the method.	*texting my friend to remember her to take her bc pill, lol.*
Mechanism of action	Tweet addresses how the contraceptive method works.	*see, i thought the iud didn't have estrogen? maybe i read it wrong. is levonorgestrel an estrogen substitute?*
Method decision making	Tweet related to making or having made a choice about which contraceptive method to use, including discontinuing, starting, or switching to a method.	*some iuds have a (minute) amount of hormones. i absolutely loved mine, thinking about getting another one this year.*
News/research/ads	Tweet provides news, research, resources, or advertisements related to the contraceptive method.	*why iuds are getting more popular with american women - health news and views -*
Politics/ethics	Tweet addressing politics or ethics around contraceptive method.	*and why did it have to be cut? extremists think an iud is an abortion*
Safety/adverse events	Tweet addresses safety of a method, including unintended adverse events or complications that occur when the contraceptive method is administered.	*1 in every 10 cases of cervical cancer is linked to taking the contraceptive pill.*
Sexual event	Tweet addressing a sexual event and how it's related to a contraceptive method.	*i've talked to a few girls who have iuds and they told me on some occasions guys can feel it*
STI	Tweet relating contraceptive method to a sexually transmitted infection.	*bitches think cause they got a birth control shot that they can just fuck anybody. bitch do yo care about catching a std??*
Uncategorized	Tweet is about a female contraceptive method but does not fit any other topic/side effect category.	*dear god, your dress is so short i could see your iud! lmao.*
**Side effects**	**Description**	**Example tweet**
Denies side effects	Tweet explicitly denying a side effect associated with contraceptive method.	*yess i don’t get any side effects from iud sometimes i forget its there. and you don’t gain weight from it either.*
Libido	Tweet relating contraceptive method use to changes in libido.	*i have done some extensive research on how the iud up’s your sexual needs.*
Mood	Tweet relating contraceptive method use to changes in mood.	*the mood swings this iud gives me are ridiculous lol*
Non-specific	Tweet broadly mentioning side effects related to contraceptive method use without mentioning a specific effect.	*do any of you have nexplanon? if so, what main side effects did you all have*
Other side effect	Tweet relating use of contraceptive method to a specific side effect not related to changes in vaginal bleeding, libido, mood, pain, skin, or weight.	*does anyone else have the iud if so does it give you nosebleeds?*
Pain (cramps)	Tweet relating contraceptive use to changes in cramping pain (not including pain with insertion or removal of IUD).	*i cramped for 3 months straight after i got my iud. it was insane.*
Pain (other/non-specified)	Tweet relating contraceptive method to pain that is not from cramps. Includes pain with insertion, removal, or administration of contraceptive.	*that birth control arm implant hurts soooo fucking bad why didn't nobody tell me this shit was painful*
Skin	Tweet relating contraceptive method use to changes in skin (i.e. acne).	*how is the depo shot in terms of hormonal acne.. also emotions, anxiety/depression ?what have you experienced*
Vaginal bleeding	Tweet relating contraceptive use to changes in vaginal bleeding or menstrual irregularities.	*girls with nexplanon, i’ve had it for almost a year now and i stopped having a period almost immediately. now i’m constantly spotting, did or does anyone else have this issue.*
Weight	Tweet relating contraceptive method use to changes in weight.	*lol well damn, have you taken that birth control shot? i know most girls gain weight after that*
**Source**	**Description**	**Example tweet**
Personal contraceptive use	Tweet shares first-person account of using or considering use of contraceptive method.	*since i have gotten my iud i’m not as emotional and don’t cry as much. which sucks because i miss having a good cry here and there*
Vicarious contraceptive use	Tweet mentions use of contraceptive method by other people (i.e. friends, partners, celebrities).	*texting my friend to remember her to take her bc pill, lol.*
News/Healthcare professional	Tweet is from a healthcare professional or news/official source.	*gynaecologists debunk myths surrounding the contraceptive pill acne, weight gain, moodiness, cancer - daily mail*
Inconclusive	There is not enough information to determine who the person posting is.	*ladies, consider getting an iud before january, some last for up to 10 yrs and anything could happen before then.*
**Information solicitation**	**Description**	**Example tweet**
Requesting information	Tweet requesting information about a contraceptive method from other users. Includes asking for factual information about, others' experiences with, and recommendations for or against the method.	*anyone recommend the contraceptive injection?*
**Advice**	**Description**	**Example tweet**
Recommending method	Tweet explicitly recommending a contraceptive method.	*look into the paragard! it’s a non-hormonal iud, good for 10 years. i got mine like 2 weeks ago and i don’t even know it’s there.*
Recommending against method	Tweet explicitly recommending against a contraceptive method.	*sorry if thats tmi but the depo shot legit ruined my life don't get it*
General advice	Tweet providing general medical advice related to the contraceptive method.	*keep using depo provera for the next five years; it may not be harmful*

*Excluding side effects

## Data Availability

The datasets used and/or analyzed during the current study are available from the corresponding author on reasonable request.
